# Disseminated Angiosarcoma With Multifocal Small‐Bowel Involvement Presenting as Suspected Small‐Bowel Bleeding

**DOI:** 10.1002/deo2.70408

**Published:** 2026-09-03

**Authors:** Mayu Tomita, Takashi Tashiro, Yoshiharu Yamaguchi, Kazunori Adachi, Masahide Ebi, Naotaka Ogasawara, Makoto Sasaki, Akana Hinokimoto, Toyonori Tsuzuki, Masanao Nakamura

**Affiliations:** ^1^ Department of Gastroenterology Aichi Medical University School of Medicine Nagakute Japan; ^2^ Department of Surgical Pathology Aichi Medical University Nagoya Japan

**Keywords:** angiosarcoma, autopsy, capsule endoscopy, double‐balloon endoscopy, small bowel bleeding

## Abstract

Angiosarcoma is a rare, aggressive vascular malignancy, and gastrointestinal involvement is uncommon. We report disseminated angiosarcoma with multifocal small‐bowel involvement presenting as suspected small‐bowel bleeding, in which small‐bowel capsule endoscopy (SBCE) and double‐balloon endoscopy (DBE) were diagnostically useful. A man in his 60s presented with recurrent melena and transfusion‐dependent anemia. SBCE showed active jejunal bleeding with multiple ulcerative lesions, and antegrade DBE revealed hemorrhagic tumors with luminal narrowing. Retrograde DBE demonstrated additional ileal and colonic lesions. Computed tomography showed small‐bowel stenosis, mesenteric dissemination, bone metastases, and soft‐tissue infiltration around an abdominal aortic stent graft. The patient died 7 weeks after transfer. Autopsy confirmed disseminated angiosarcoma involving the small intestine and mesentery, with the greatest tumor burden around the aortic graft, suggesting a possible periaortic origin. This case highlights the complementary roles of SBCE and DBE and provides rare clinicopathological correlation with autopsy findings.

## 1 Introduction

Angiosarcoma is a highly aggressive vascular endothelial malignancy accounting for less than 2% of soft tissue sarcomas [1, 2]. Gastrointestinal involvement, particularly of the small bowel, is rare and may present with anemia or gastrointestinal bleeding [2, 3, 4, 5].

Although small‐bowel capsule endoscopy (SBCE) and double‐balloon endoscopy (DBE) have improved evaluation of suspected small‐bowel bleeding (SSBB), the endoscopic features of small‐bowel angiosarcoma remain poorly characterized. We report a case in which SBCE and DBE enabled antemortem diagnosis, and autopsy provided clinicopathological correlation.

## 2 Case Report

### 2.1 Clinical Presentation

A man in his 60s was referred for suspected small‐bowel bleeding. His history included thoracoabdominal aortic dissection treated with vascular graft implantation, chronic heart failure, chronic kidney disease, hypertension, dyslipidemia, and obstructive sleep apnea.

Several weeks earlier, he had developed fatigue and melena. Despite endoscopic hemostasis for duodenal bleeding, anemia progressed, and bleeding scintigraphy suggested small‐intestinal hemorrhage.

At presentation, he was taking prednisolone (25 mg/day) for unexplained inflammatory findings with elevated IgG4, lansoprazole, rosuvastatin, and magnesium oxide; he was not receiving anticoagulants, antiplatelet agents, or NSAIDs.

Laboratory tests showed hemoglobin 7.0 g/dL, white blood cells 17,700/mm^3^, total protein 5.0 g/dL, albumin 2.0 g/dL, C‐reactive protein 6.6 mg/dL, IgG 968 mg/dL, and mildly elevated IgG4 170 mg/dL. CEA (1.7 ng/mL) and CA19‐9 (4 U/mL) were not elevated.

Emergency upper endoscopy showed multiple small oozing duodenal erosions, which were insufficient to explain the persistent melena and severe anemia.

### 2.2 SBCE Findings

**FIGURE 1:**
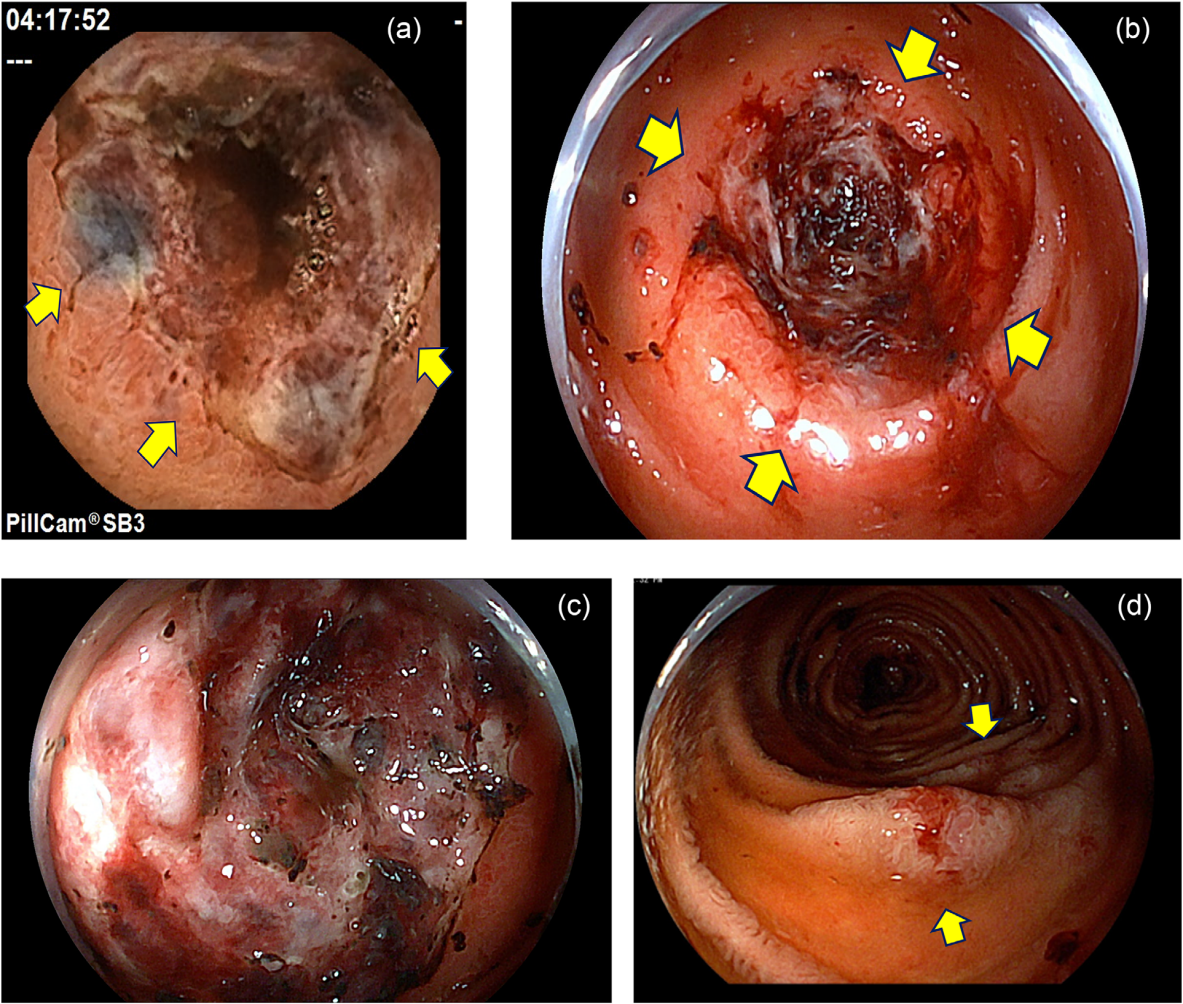
Endoscopic findings. (a) Small‐bowel capsule endoscopy showing active bleeding from an irregular circumferential ulcerative lesion in the mid‐jejunum (arrows). (b) Antegrade double‐balloon endoscopy (DBE) showing a friable ulcerated tumor with surrounding mucosal elevation and luminal narrowing in the jejunum (arrows). (c) Close‐up view showing an irregular indurated ulcer base with active oozing. (d) Retrograde DBE showing multiple elevated ileal lesions with surface oozing (arrows).

The patient had no abdominal pain, distension, or other obstructive symptoms. Because bleeding scintigraphy suggested a small‐bowel source and rapid localization of ongoing bleeding was prioritized, SBCE was performed before computed tomography (CT). The patient had not undergone regular CT surveillance immediately before referral. SBCE demonstrated active jejunal bleeding with multiple ulcerative and hemorrhagic lesions (Figure 1a; Video S1).

### 2.3 DBE Findings

Antegrade DBE revealed multiple hemorrhagic elevated jejunal lesions with severe luminal narrowing (Figure 1b, c). A patency capsule had not been used because there were no obstructive symptoms. The capsule endoscope was temporarily retained at the stenosis and was retrieved during antegrade DBE. Gastrografin enterography was not performed because ongoing transfusion‐dependent bleeding raised concern that additional intraluminal pressure and mechanical stimulation might exacerbate hemorrhage. Biopsy specimens showed highly atypical malignant cells forming irregular vascular channels, suggesting a malignant vascular neoplasm.

Retrograde DBE revealed additional friable elevated lesions in the distal ileum and ascending colon (Figure 1d).

**FIGURE 2:**
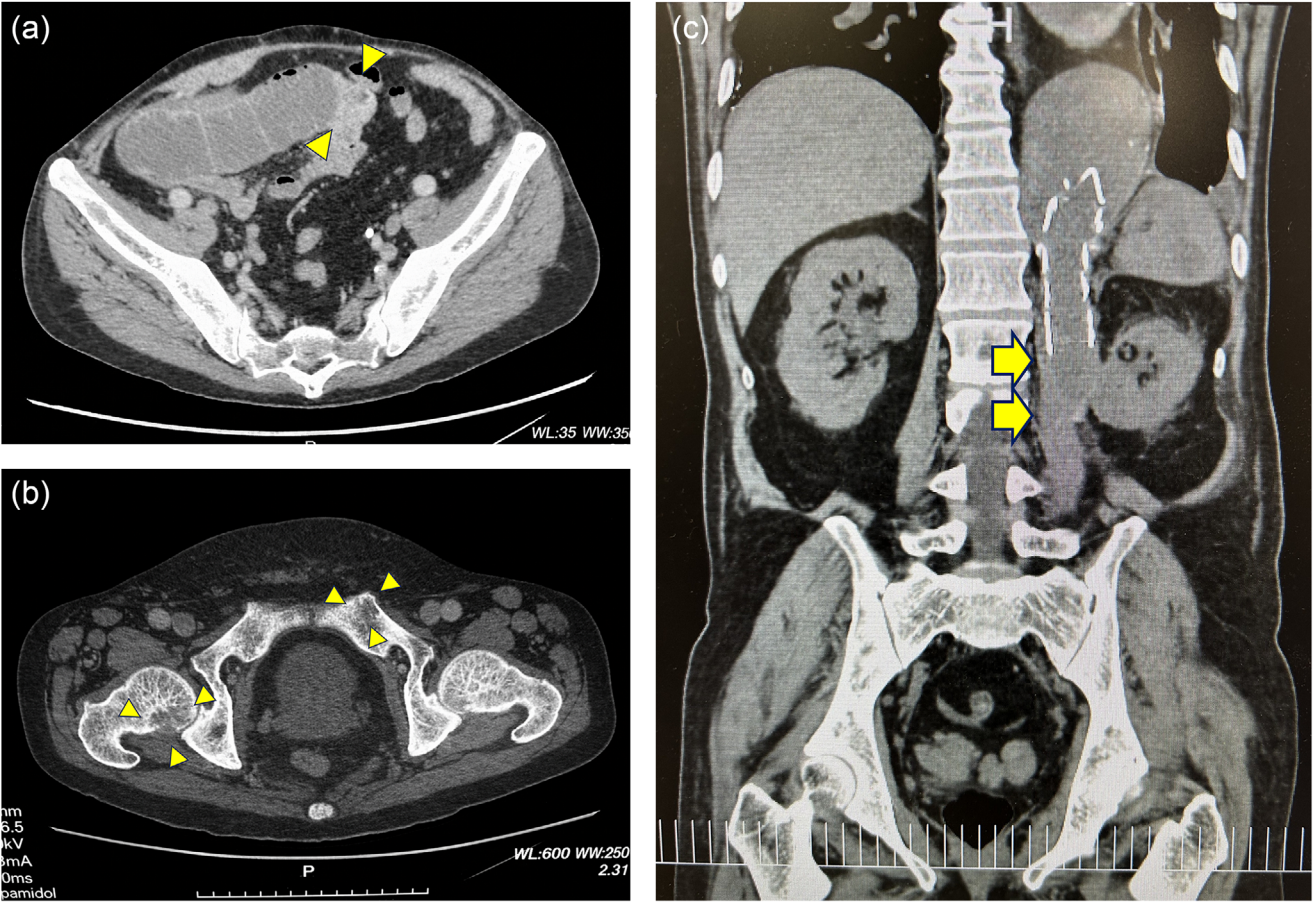
Computed tomography (CT) scan. (a) Contrast‐enhanced CT showing focal jejunal wall thickening with associated luminal stenosis at the bleeding site (arrowheads). (b) CT bone window demonstrating multiple suspected bone metastases (arrowheads). (c) Abdominal aortic graft with soft‐tissue infiltration (arrows).

Contrast‐enhanced CT demonstrated small‐bowel stenosis, mesenteric dissemination, multiple osseous metastases, bladder nodules, and soft‐tissue infiltration around the abdominal aortic stent graft (Figure 2a–c).

### 2.4 Clinical Course

Given the advanced disease, poor performance status, and comorbidities, neither surgery nor systemic chemotherapy was considered appropriate, and best supportive care was provided. The patient died approximately 7 weeks after transfer.

### 2.5 Autopsy and Histopathological Findings

**FIGURE 3:**
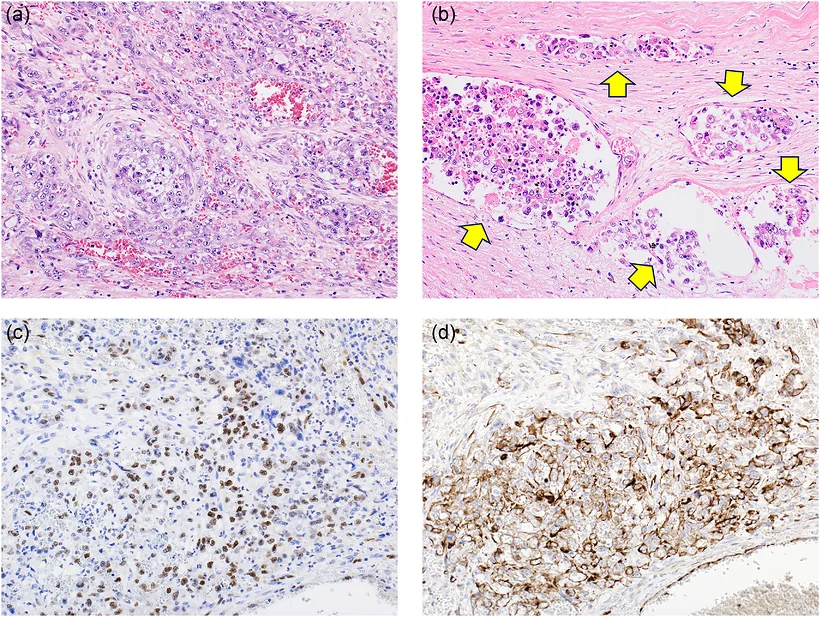
Histopathological findings of angiosarcoma. (a) Hematoxylin and Eosin (H&E) staining showing sheets of atypical epithelioid tumor cells with enlarged nuclei and prominent nucleoli. (b) H&E staining showing tumor cells within vascular lumina of the aortic wall, indicating lymphovascular invasion (arrows). (c) Immunohistochemistry showing strong nuclear expression of ERG in tumor cells. (d) Tumor cells showing strong expression of pancytokeratin (AE1/AE3 + CAM5.2).

**FIGURE 4:**
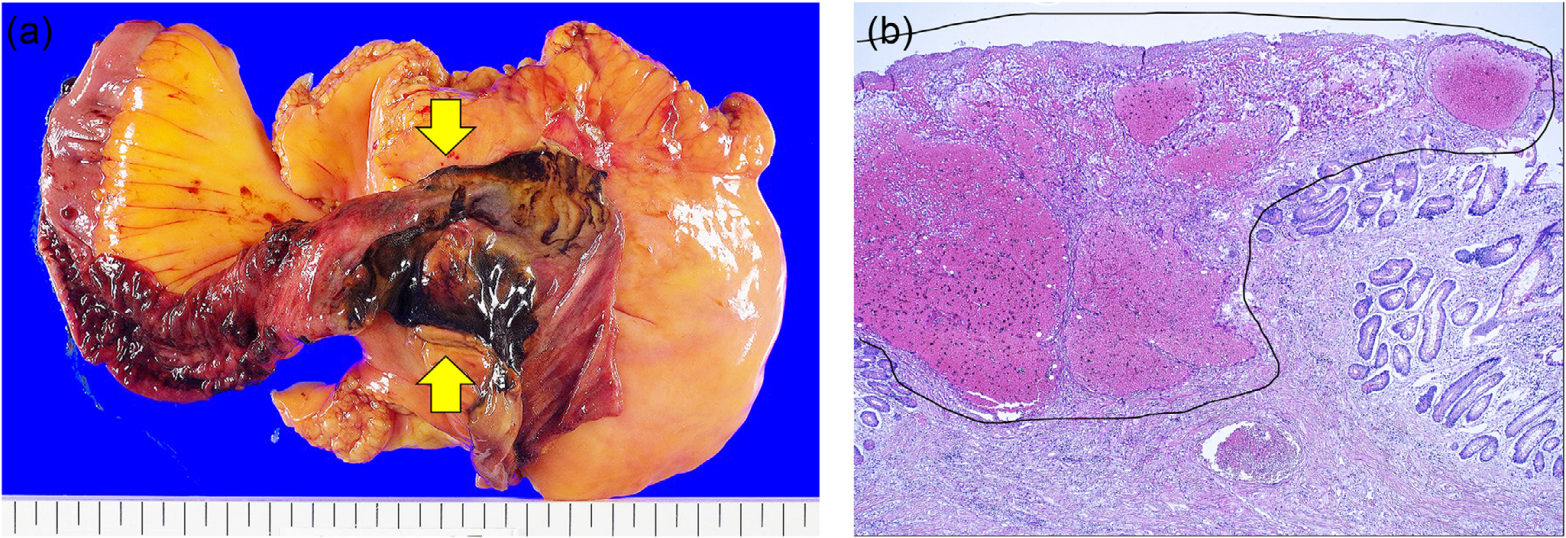
Jejunal involvement at autopsy. (a) Gross specimen showing jejunal tumors associated with intestinal stenosis (arrows). (b) Histological section showing invasive angiosarcoma infiltrating the intestinal wall (Hematoxylin and Eosin stain); the area outlined in black indicates tumor involvement.

Autopsy revealed numerous hemorrhagic nodules throughout the mesentery and small intestine. Histology showed infiltrative atypical epithelioid and spindle cells forming irregular vascular channels. Tumor cells were positive for ERG, CD31, CD34, AE1/AE3, and CAM5.2 (Figure 3a–d), confirming angiosarcoma. The greatest tumor burden involved the abdominal aortic graft and adjacent retroperitoneum (Figure S1), suggesting a possible periaortic origin with secondary dissemination to the mesentery and small intestine (Figure 4a, b).

## 3 Discussion

Few reports have documented small‐bowel angiosarcoma from SBCE detection and DBE biopsy to autopsy confirmation. In our patient, SBCE localized active jejunal bleeding, whereas DBE defined multifocal hemorrhagic stenotic lesions and enabled tissue diagnosis.

Reported endoscopic appearances include hemorrhagic polyps, reddish nodules, submucosal tumor‐like lesions, ulcerative masses, and friable vascular tumors [4, 7]. Thus, combining SBCE for noninvasive localization with DBE for direct assessment and biopsy may be particularly useful when conventional endoscopy cannot explain the severity of bleeding [6, 7].

Another notable feature was the possible association with the abdominal aortic stent graft. Angiosarcoma associated with vascular prostheses or endovascular grafts is exceedingly rare [3, 8]. Chronic injury, foreign‐body reaction, inflammation, fibrosis, and vascular proliferation have been proposed as contributing mechanisms, although causality in humans remains unproven [9, 10].

Vascular graft‐associated angiosarcoma is aggressive, often presenting with local invasion or distant dissemination and poor prognosis [3, 8, 9, 10]. In this case, the greatest tumor burden surrounded the graft, whereas gastrointestinal lesions were multifocal and disseminated.

Although the primary site could not be established definitively, the distribution strongly suggested a periaortic origin with spread to the mesentery and small bowel.

In conclusion, this case demonstrates the complementary diagnostic value of SBCE and DBE in SSBB and provides rare endoscopic‐pathological correlation in disseminated angiosarcoma with multifocal small‐bowel involvement.

## Author Contributions

**Mayu Tomita**: writing – original draft, conceptualization, investigation, and data curation. **Takashi Tashiro**: investigation. **Yoshiharu Yamaguchi**: investigation. **Kazunori Adachi**: investigation. **Masahide Ebi**: investigation. **Naotaka Ogasawara**: conceptualization. **Makoto Sasaki**: conceptualization. **Akana Hinokimoto**: supervision. **Toyonori Tsuzuki**: supervision. **Masanao Nakamura**: conceptualization and writing – review and editing. All authors have read and approved the final manuscript.

## Funding

The authors received no funding from external sources for this manuscript. The authors have nothing to report.

## Ethics Statement

This study was conducted in accordance with the ethical standards laid down in the 1964 Declaration of Helsinki and its later amendments.

## Consent

Written informed consent for publication of this case report and accompanying images was obtained from the patient's wife because the patient was deceased.

## Conflicts of Interest

The authors declare no conflict of interest.

## Supporting information




**FILE S1**: deo270408‐sup‐0001‐FigureS1.tif

**Video S1**: Small‐bowel capsule endoscopy showing active jejunal bleeding with the ulcerative lesion.

**FILE S3**: deo270408‐sup‐0003‐SuppMat.docx

## Data Availability

The data that support the findings of this study are available from the corresponding author upon reasonable request.
