# Supplementary material for: Disseminated Angiosarcoma With Multifocal Small‐Bowel Involvement Presenting as Suspected Small‐Bowel Bleeding
Source: DEN Open. 2026 Sep 3;7(1):e70408. doi: 10.1002/deo2.70408 (PMC13540639; doi:10.1002/deo2.70408)
Supplement: Supplementary file 1 [file DEO2-7-e70408-s003.docx]

Supporting Information Legend

Figure S1. Gross autopsy specimen showing extensive hemorrhagic tumor involvement surrounding the abdominal aortic stent graft and adjacent retroperitoneal tissue.

Video legend: Small-bowel capsule endoscopy demonstrating active bleeding from an ulcerative lesion in the jejunum.
